# The usefulness of the retinal sensitivity measurement with a microperimetry for predicting the visual prognosis of branch retinal vein occlusion with macular edema

**DOI:** 10.1007/s00417-020-04759-9

**Published:** 2020-05-26

**Authors:** Ryosuke Fujino, Ryo Asaoka, Shuichiro Aoki, Aya Sugiura, Mari Kusakabe, Kimiko Asano-Shimizu, Yoko Nomura, Aya Aoki, Yohei Hashimoto, Keiko Azuma, Tatsuya Inoue, Ryo Obata

**Affiliations:** 1 Department of Ophthalmology, The University of Tokyo, 7-3-1 Hongo, Bunkyo-ku, Tokyo 113-8655 Japan; 2 Asahi General Hospital, Chiba, Japan; 3 Department of Ophthalmology and Micro-technology, Yokohama City University, Yokohama, Kanagawa Japan

**Keywords:** Branch retinal vein occlusion, Macular edema, Retinal sensitivity, MP-3

## Abstract

**Purpose:**

To evaluate the usefulness of the retinal sensitivity in branch retinal vein occlusion (BVO) with macular edema (ME) following the anti-vascular endothelial growth factor (anti-VEGF) treatment.

**Methods:**

Best-corrected visual acuity (BCVA), microperimetry, and optical coherence tomography (OCT) measurements were carried out in 20 patients with BVO with ME, at baseline and 1 month after the anti-VEGF treatment. The relationships among BCVA, mean retinal sensitivity (MS), macular volume (MV), central retinal thickness (CRT), integrity of ellipsoid zone (EZ), mean retinal sensitivity in the most affected quadrant (qMS), and macular volume in the most affected quadrant (qMV) were investigated. In addition, the relationships among the change in BCVA at 1 month (ΔBCVA1m), mean sensitivity in the most affected quadrant at 1 month (ΔqMS1m), MV in the most affected quadrant at 1 month (ΔqMV1m), and CRT at 1 month (ΔCRT1m) were analyzed. The optimal model for BCVA at 3 months after the treatment (BCVA3m) was identified.

**Results:**

There was not a significant difference in BCVA (paired Wilcoxon test, *p* = 0.058) between at baseline and after the treatment, but there were significant differences in MS, MV, CRT, qMS, and qMV (*p* < 0.05). There was a significant relationship between ΔqMS1m and ΔMV1m, ΔCRT1m, and ΔqMV1m, respectively. ΔMS1m or ΔqMS1m and BCVA at baseline and ΔBCVA1m were selected as explanatory variables in the optimal model for BCVA3m.

**Conclusion:**

Retinal sensitivity was related to retinal structure, whereas this was not the case with BCVA. In addition, retinal sensitivity was useful to predict BCVA after anti-VEGF therapy.

## Introduction

Retinal vein occlusions (RVOs) are one of the most common retinal vascular diseases [1]. In RVOs, such as branch RVO (BVO), patient’s sights are threatened by various associated complications, including macular edema (ME). ME in RVOs is usually treated with the intravitreal injection of anti-vascular endothelial growth factor (anti-VEGF) agents as the first-line therapy [2–6]. The identification and quantitative evaluation of ME have been greatly enhanced with the development of optical coherence tomography (OCT) [2], and the treatment strategies are usually decided basing on both of visual acuity (VA) and OCT-measured ME thickness after the anti-VEGF treatment. It should be noted that BCVA mainly reflects visual function only around fovea, whereas ME impairs visual function not only at fovea but also in a surrounding area. Agreeing with this, previous studies suggested that retinal sensitivity measured with perimetry is associated with OCT-measured macular structural change, using the Humphrey Field Analyzer (HFA, Carl-Zeiss Meditec AG, Dublin, CA, USA) [3] and the MP1 Microperimeter (Nidek CO., LTD, Aichi, Japan) [7] in eyes with RVO with ME. In addition, the improvement of visual functional (BCVA and retinal sensitivity) is significantly associated with morphological (OCT-measured retinal thickness) improvement in RVO and ME eyes after the intravitreal anti-VEGF treatment [7]. Moreover, a recent study suggested that the retinal sensitivity measured with a microperimetry of MAIA (Center Vue, Padova, Italy) 1 day after the anti-VEGF treatment was significantly related with BCVA at 1 month in eyes with BVO with ME, suggesting the potential usefulness of retinal sensitivity in predicting the BCVA prognosis [8], although it was not investigated whether it is useful to measure retinal sensitivity, in addition to BCVA.

The MP-3 microperimeter (Nidek CO., LTD, Aichi, Japan) is a relatively new perimetry instrument. In contrast to its predecessor of the MP-1, the MP-3 has a wide dynamic range (between 0 and 34 dB) on the background luminance of 31.5 ASB which is identical to that with the HFA. Furthermore, in this microperimeter, the target light is projected onto the retina directly, rather than on a screen like in the HFA, which enables automatic tracking of retina. As a result, the exact same location can be stimulated in repeated target presentations. We previously reported visual retinal sensitivity measured with MP-3 has a better test-retest reproducibility than HFA in patients with retinitis pigmentosa (RP) [9] and glaucoma [10]. This advantage of MP-3 would be more pronounced in the eyes with ME, because the damage in central retina would hamper the fixation of an eye during the VF measurement. Thus, the purpose of the current study was to investigate whether it is advantageous to measure the retinal sensitivity using MP-3, in addition to BCVA, to predict the prognosis of BCVA, in eyes with BVO with ME following the anti-VEGF treatment.

## Methods

This was a retrospective study and the procedures were approved by the Research Ethics Committee of the Graduate School of Medicine and Faculty of Medicine at The University of Tokyo (#3770). Written consent was given by patients for their information to be stored in the hospital database and used for research. This study was performed according to the tenets of the Declaration of Helsinki.

### Subjects

This study included 20 eyes of 20 consecutive BVO patients (9 males and 11 females). All patients were major BVO not macular BVO. Five patients underwent 0.5 mg of ranibizumab intravitreal injection whereas 15 patients were given the injection of 2 mg of aflibercept. Seventeen eyes had already received anti-VEGF treatments 3.95 times (standard deviation, SD = 3.4) on average prior to the initiation of the current study.

Each patient had received one injection followed by the pro re nata phase (1 + PRN regimen) when the central macular thickness was greater than 250 μm.

All patients enrolled in the study fulfilled the following criteria: (1) BVO was the only disease-causing VF damage; (2) no other treatments for ME, such as laser photocoagulation, intraocular surgery, and intravitreal steroid injection, were performed prior to the initiation of the current study and also during the follow-up period; (3) refractive error between − 6 and + 6 diopter (D); (4) no obvious ischemic changes in macula confirmed using OCT angiography (Heidelberg Engineering, Heidelberg, Germany).

BCVAs (LogMAR) were measured at baseline and 1, 3, and 12 months after the anti-VEGF treatment (BCVAbase, BCVA1m, BCVA3m, and BCVA12m, respectively).

### Optical coherence tomography measurement

**Fig. 1:**
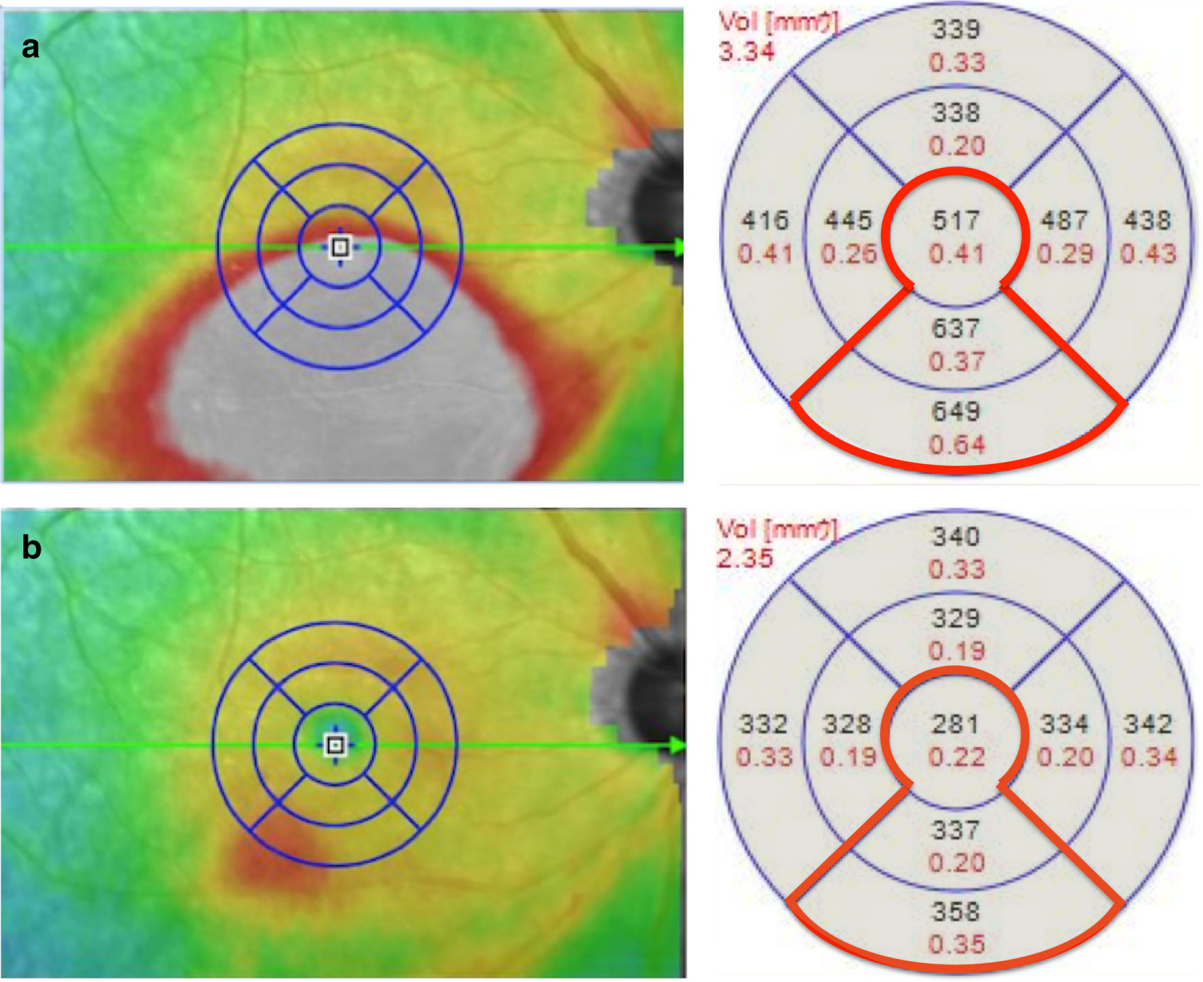
OCT images with MV and qMV at baseline and 1 month after anti-VEGF treatment in a representative case (64 years old, female). The OCT images at baseline and 1 month after anti-VEGF treatment are indicated in **a** and **b**, respectively. The diameter of the center, inner, and outer ring was 1, 2, and 3 mm, respectively. MV was the total macular volume in the central 3 mm circle. qMV was the volume inner red line in this case. MV (macular volume) and qMV (macular volume) in the quadrant correspond to the most predominantly affected

Spectral domain (SD) OCT measurement was performed using the Spectralis OCT at baseline and 1 month after the anti-VEGF treatment. The OCT images consisted of line scans (horizontal and vertical B-scans), raster scans, and topographic mapping. Line scans were created by taking the average of 100 B-scans (768 A-scans per B-scan) within 30°. The raster scan was performed using 25 B-scans (768 A-scans per B-scan) of a 30° × 20° area. Then, the average of the central retinal thickness (CRTbase: at baseline and CRT1m: at 1 month after anti-VEGF treatment) and macular volume (MVbase: at baseline and MV1m: at 1 month after anti-VEGF treatment) were calculated. Furthermore, MV in the most predominantly affected quadrant was calculated (qMVbase: at baseline and qMV1m: at 1 month after anti-VEGF treatment). The most predominantly affected quadrant was initially identified by an examiner (R.F.), followed by verification by an independent examiner (T.I.). If the second estimator did not agree with the first examiner, a panel discussion (R.F., T.I., K.A., R.O.) was held to draw a conclusion. The OCT images of a representative case reflecting the MV and qMV at baseline and 1 month after anti-VEGF treatment are shown in Fig. 1. The differences of these OCT thicknesses between baseline and 1 month after anti-VEGF treatment were calculated (ΔCRT1m, ΔMV1m, and ΔqMV1m, respectively).

### MP-3 measurement

**Fig. 2:**
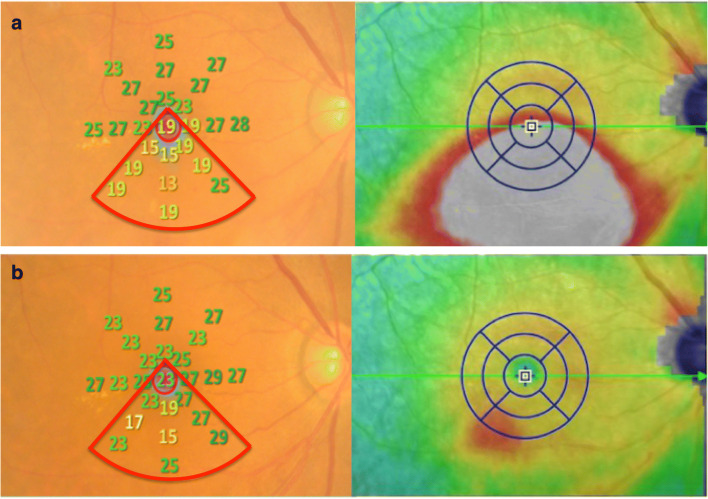
The MP-3 images superimposed onto OCT image at baseline and 1 month after anti-VEGF treatment in a representative case (64 years old, female). The MP-3 images baseline and 1 month after anti-VEGF treatment are indicated in **a** and **b**, respectively. A total of 25 stimulus locations covering the central 6° field were tested. MS was calculated as the average of the sensitivities at 25 locations. The qMS was calculated as the average of the sensitivities at 10 locations inner red line in this case because the most predominantly affected quadrant determined with OCT image was underside. OCT optical coherence tomography, VEGF anti-vascular endothelial growth factor, MS mean retinal sensitivity, qMS mean sensitivity in the quadrant corresponds to the most predominantly affected on optical coherence tomography

MP-3 measurement was conducted at baseline and 1 month after anti-VEGF treatment. All patients had a pupil size larger than 4 mm in diameter, as required for the MP-3 measurement. The MP-3 measurement was carried out using the 4–2 full threshold staircase strategy using the standard Goldmann III stimulus size on the background luminance of 31.4 ASB. The maximum luminance of the MP-3 is 10,000 ASB, which results in the stimulus dynamic range between 0 and 34 dB. Eccentric circle was drawn at 2, 4, and 6° from fovea and eight test points were allocated on each circle, in addition to fovea (Fig. 2: MP-3 images superimposed onto OCT images in a representative case). Only reliable VFs, defined as fixation loss (FL) rate < 20% and a false-positive (FP) rate < 15%, were used in analyses.

The mean retinal sensitivities of all 25 test points at baseline and 1 month after the anti-VEGF treatment were calculated (MSbase: baseline and MS1m: 1 month after anti-VEGF treatment, respectively). In addition, the mean sensitivity in the quadrant corresponds to qMV was also calculated (qMSbase: baseline and qMS1m: 1 month after anti-VEGF treatment, respectively). The differences of these values between baseline and 1 month after anti-VEGF treatment were calculated (ΔMS1m and ΔqMS1m).

### Statistical analysis

Comparisons of numerical values between two groups were performed using the paired Wilcoxon test. The relationships between (1) CRTbase and the variables of BCVAbase, MSbase, and qMSbase; (2) CRT1m and the variables of BCVA1m, MS1m, and qMS1m; (3) MVbase and the variables of BCVAbase, MSbase, and qMSbase; (4) MV1m and the variables of BCVA1m, MS1m, and qMS1m; (5) qMVbase and the variables of BCVAbase, MSbase, and qMSbase; (6) qMV1m and the variables of BCVA1m, MS1m, and qMS1m; (7) ΔCRT1m and the variables of ΔBCVA1m, ΔMS1m, and ΔqMS1m; (8) ΔMV1m and the variables of ΔBCVA1m, ΔMS1m, and ΔqMS1m; and (9) ΔqMV1m and the variables of ΔBCVA1m, ΔMS1m, and ΔqMS1m were investigated using the linear regression model.

Also, the relationship between BCVA3m and the variables of BCVAbase, ΔBCVA1m, MSbase, ΔMS1m, integrity of EZ, the frequency of the injection of anti-VEGF, and age was investigated using the multivariate linear regression model. Then, the optimal linear model was selected according to the second-order bias-corrected Akaike Information Criterion (AICc) index. Similar analysis was carried out using the variables of BCVAbase, ΔBCVA1m, qMSbase (instead of MSbase), ΔqMS1m (instead of ΔMS1m), integrity of EZ, the frequency of the injection of anti-VEGF, and age. The AICc is the corrected form of the common statistical measure of AIC. AICc gives an accurate estimation even when the sample size is small [11]. In a multivariate regression model, degrees of freedom decreases as the number of variables increases; hence, it is recommended to use model selection methods to improve the model by removing redundant variables [12, 13]. Any magnitude of reduction in AICc suggests an improvement of the model, and the probability that one particular model is the model that minimizes “information loss” can be calculated, when there are *n* candidate models and the AICc values of those models are AIC1, AIC2, AIC3, ..., AIC*n*. If AICmin is the minimum of these values, then exp.((AICmin − AIC*i*)/2) describes the relative probability that the *i*th model minimizes the information loss (i.e., the “optimal model”) [14] Relative probabilities were calculated among all candidate models. All statistical analyses were performed using the statistical programming language “R” (R version 3.1.3; e foundation for Statistical Computing, Vienna, Austria).

## Results

Table 1 shows the subjects’ demographics. The mean (± standard deviation: SD) age was 68.9 ± 7.2 (range 52 to 79) years, and 9 patients were male and 11 patients were female. BCVAbase was 0.13 ± 0.21 (− 0.079 to 0.7), MSbase was 22.7 ± 2.2 (17.2 to 26.7) dB, and MVbase was 2.8 ± 0.37 (2.3 to 3.7) mm^3^. CRTbase was 330.6 ± 135.2 (164 to 622) μm. qMSbase was 19.6 ± 3.3 (12.8 to 26) dB. qMVbase was 1.0 ± 0.24 (0.73 to 1.6) mm^3^. Among 20 eyes, qMV was in the superior quadrant in 8 eyes, whereas it was in the inferior quadrant in 12 eyes.

**Table 1:** Subjects’ demographics

Variables	Value	*p* value
Age, years old, mean ± sd	68.9 ± 7.2	-
Gender, male:female	9:11	-
Times of anti-VEGF treatment	3.95 ± 3.4	
BCVAbase, LogMAR, mean ± sd	0.13 ± 0.21	-
BCVA1m, LogMAR, mean ± sd	0.11 ± 0.27	0.058
BCVA3m, LogMAR, mean ± sd	0.059 ± 0.18	0.033
BCVA1Y, LogMAR, mean ± sd	0.036 ± 0.15	0.002
MSbase, dB, mean ± sd	22.7 ± 2.2	-
MS1m, dB, mean ± sd	24.5 ± 2.0	0.0017
MVbase, mm^3^, mean ± sd	2.8 ± 0.37	-
MV1m, mm^3^, mean ± sd	2.3 ± 0.15	< 0.001
CRTbase, μm, mean ± sd	330.6 ± 135.2	-
CRT1m, μm, mean ± sd	197.4 ± 30.8	< 0.001
qMSbase, dB, mean ± sd	19.6 ± 3.3	-
qMS1m, dB, mean ± sd	27.2 ± 2.7	< 0.001
qMVbase, mm^3^, mean ± sd	1.0 ± 0.24	-
qMV1m, mm^3^, mean ± sd	0.74 ± 0.063	< 0.001

*BCVA*, best-corrected visual acuity; *MS*, mean retinal sensitivity; *MV*, macular volume; *CRT*, central retinal thickness; *qMS*, mean sensitivity in the quadrant corresponds to the most predominantly affected on optical coherence tomography; *qMV*, macular volume in the quadrant corresponds to the most predominantly affected; *sd*, standard deviation

*p* values were calculated by comparing each variable between before and after the treatment

There was not a significant difference in BCVAbase and BCVA1m (*p* = 0.058, paired Wilcoxon test). However, significant differences were observed between BCVAbase and BCVA3m (*p* = 0.033) and between BCVAbase and BCVA12m (*p* = 0.002). On the other hand, there was a significant difference between MSbase and MS1m, MVbase and MV1m, CRTbase and CRT1m, qMSbase and qMS1m, and qMVbase and qMV1m (paired Wilcoxon test, *p* = 0.0017, < 0.001, < 0.001, < 0.001 and < 0.001, respectively).

There was a significant negative relationship between BCVAbase and MSbase (coefficient = − 0.050, *p* = 0.014, linear regression) and between BCVAbase and qMSbase (coefficient = − 0.032, *p* = 0.021, linear regression). Although a significant correlation was not observed between BCVA1m and MS1m (*p* = 0.10, linear regression), there was a significant relationship between ΔBCVA1m and ΔMS1m (coefficient = − 0.038, *p* = 0.048, linear regression) and ΔBCVA1m and ΔqMS1m (coefficient = − 0.028, *p* = 0.048, linear regression).

Table 2 shows the relationships between the values of CRT and MV and the variables of BCVAbase, MSbase, qMSbase, and age. qMSbase was significantly related to CRTbase (coefficient = − 52.7, *p* = 0.014, linear regression). The relationships between CRT1m and the variables of BCVA1m, MS1m, qMS1m, and age are also shown in Table 2. There was no parameter significantly related to CRT1m. qMSbase was significantly related to MVbase (coefficient = − 0.14, *p* = 0.0093, linear regression). There was no parameter significantly related to MV1m.

**Table 2:** The relationship between CRT and BCVA, MS, qMS, and age, and between MV and BCVA, MS, qMS, and age, before and after the treatment

Multivariate analysis				
Independent variable	Dependent variable	Correlation coefficient	Standard error	*p* value
CRTbase	BCVAbase	13.1	158	0.93
	MSbase	54.4	28.3	0.074
	qMSbase	− 52.7	18.9	*0.014*
	Age	− 4.25	4.14	0.32
CRT1m	BCVA1m	− 22.9	44.1	0.61
	MS1m	8.73	10.4	0.41
	qMS1m	− 10.8	7.63	0.18
	Age	− 0.65	1.13	0.57
MVbase	BCVAbase	0.0069	0.38	0.99
	MSbase	0.1	0.069	0.15
	qMSbase	− 0.14	0.046	*0.0093*
	Age	− 0.019	0.01	0.083
MV1m	BCVA1m	− 0.074	0.23	0.75
	MS1m	0.047	0.054	0.4
	qMS1m	− 0.046	0.04	0.27
	Age	− 0.0036	0.006	0.56

*BCVA*, best-corrected visual acuity; *MS*, mean retinal sensitivity; *qMS*, mean sensitivity in the quadrant corresponds to the most predominantly affected on optical coherence tomography; *MV*, macular volume; *CRT*, central retinal thickness

Italic *p* values indicated significant

As shown in Table 3, qMSbase was significantly related to qMVbase (coefficient = − 0.077, *p* = 0.033, linear regression). There was no parameter significantly related to qMV1m.

**Table 3:** The relationship between qMV and BCVA, MS, qMS and age, before and after the treatment

Multivariate analysis				
Independent variable	Dependent variable	Correlation coefficient	Standard error	*p* value
qMVbase	BCVAbase	0.059	0.27	0.83
	MSbase	0.059	0.049	0.25
	qMSbase	− 0.077	0.033	*0.033*
	Age	− 0.011	0.0072	0.14
qMV1m	BCVA1m	− 0.03	0.099	0.77
	MS1m	0.0053	0.023	0.82
	qMS1m	− 0.0038	0.017	0.83
	Age	− 0.00027	0.0026	0.92

*BCVA*, best-corrected visual acuity; *MS*, mean retinal sensitivity; *qMS*, mean sensitivity in the quadrant corresponds to the most predominantly affected on optical coherence tomography; *qMV*, macular volume in the quadrant corresponds to the most predominantly affected

Italic *p* values indicated significant

**Fig. 3:**
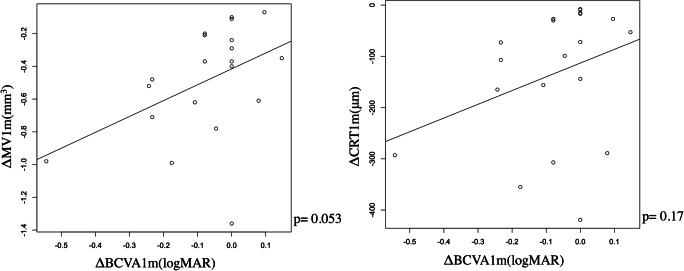
The relationships between ΔBCVA1m and ΔMV1m, and between ΔBCVA1m and ΔCRT1m. ΔBCVA1m was not significantly correlated with ΔMV1m (left panel, *p* = 0.053) and ΔCRT1m (right panel, *p* = 0.17). ΔBCVA1m: difference of best-corrected visual acuity before and 1 month after treatment, ΔMV1m: difference of macular volume before and 1 month after treatment, ΔCRT1m: difference of central retinal thickness before and 1 month after treatment

Figure 3 shows the relationships between ΔBCVA1m and ΔMV1m, and between ΔBCVA1m and ΔCRT1m; significant relationship was not observed (*p* = 0.053 and 0.17, linear regression, respectively). Similar result was obtained with the multivariable regression analysis (Table 4).

**Table 4:** . The relationship between ΔCRT1m and ΔBCVA1m, ΔMS1m, ΔqMS1m, and age, between ΔMV1m and ΔBCVA1m, ΔMS1m, ΔqMS1m, and age, and between ΔqMV1m and ΔBCVA1m, ΔMS1m, ΔqMS1m, and age

Multivariate analysis				
Independent variable	Dependent variable	Correlation coefficient	Standard error	*p* value
ΔCRT1m	ΔBCVA1m	120	180	0.52
	ΔMS1m	59.6	28.4	0.053
	ΔqMS1m	− 62.7	20.7	*0.0084*
	Age	2.79	3.40	0.42
ΔMV1m	ΔBCVA1m	0.49	0.43	0.27
	ΔMS1m	0.13	0.067	0.07
	ΔqMS1m	− 0.16	0.049	*0.0055*
	Age	0.0078	0.008	0.35
ΔqMV1m	ΔBCVA1m	0.28	0.3	0.36
	ΔMS1m	0.081	0.047	0.1
	ΔqMS1m	− 0.099	0.034	*0.011*
	Age	0.006	0.0056	0.3

*ΔBCVA*, difference of best-corrected visual acuity before and after treatment; *ΔMS*, difference of mean retinal sensitivity; *ΔMV*, difference of macular volume; *ΔCRT*, difference of central retinal thickness; *ΔqMS*, difference of mean sensitivity in the quadrant corresponds to the most predominantly affected on optical coherence tomography; *ΔqMV*, difference of macular volume in the quadrant corresponds to the most predominantly affected

Italic *p* values indicated significant

**Fig. 4:**
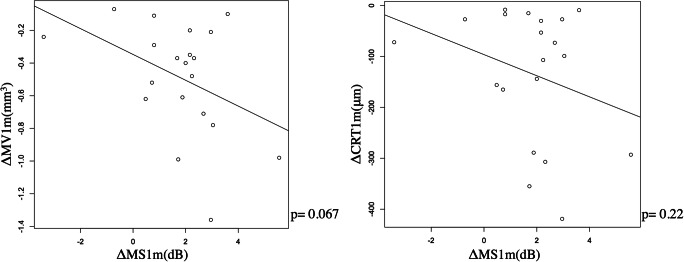
The relationships between ΔMS1m and ΔMV1m, and between ΔMS1m and ΔCRT1m. ΔMS1m was not significantly correlated with ΔMV1m (left panel, *p* = 0.067) and ΔCRT1m (right panel, *p* = 0.22). ΔMS1m: difference of mean retinal sensitivity before and 1 month after treatment, ΔMV1m: difference of macular volume before and 1 month after treatment, ΔCRT1m: difference of central retinal thickness before and 1 month after treatment

**Fig. 5:**
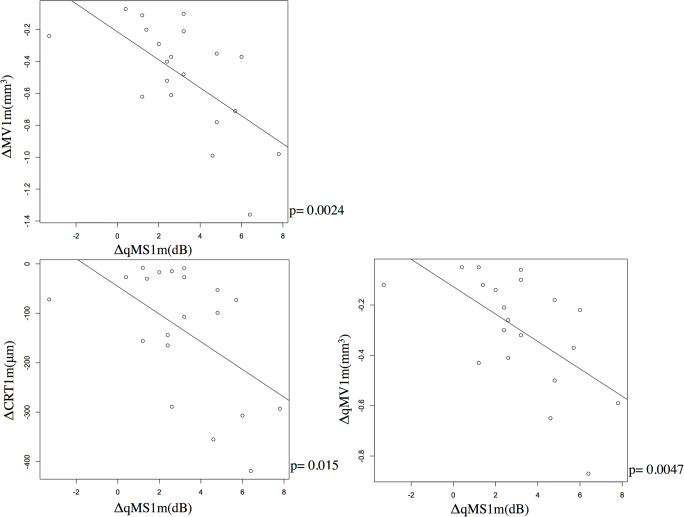
The relationships between ΔqMS1m and ΔMV1m, ΔqMS1m and △CRT_1m_, and ΔqMS1m and ΔqMV1m. ΔqMS1m was significantly correlated with ΔMV1m, ΔCRT1m, and ΔqMV1m (*p* = 0.0024, 0.015, and 0.0047, respectively). ΔqMS1m: difference of mean sensitivity in the quadrant corresponds to the most predominantly affected on optical coherence tomography before and 1 month after treatment, ΔMV1m: difference of macular volume before and 1 month after treatment, ΔCRT1m: difference of central retinal thickness before and 1 month after treatment, ΔqMV1m: difference of macular volume in the quadrant corresponds to the most predominantly affected before and 1 month after treatment

As shown in Fig. 4, there was no significant relationship between ΔMS1m and ΔMV1m, and between ΔMS1m and ΔCRT1m (*p* = 0.067 and 0.22, respectively, linear regression). Similar result was obtained with the multivariable regression analysis (Table 4). In contrast, as shown in Fig. 5, significant relationship was observed between ΔqMS1m and ΔMV1m (ΔqMS = 0.85–4.7 x ΔMV, *p* = 0.0024), between ΔqMS1m and ΔCRT 1 m (ΔqMS = 1.76–0.01 x ΔCRT, *p* = 0.015), and between ΔqMS1m and ΔqMV1m (ΔqMS = 1.13–6.72 x ΔqMV, *p* = 0.0047). Similar result was obtained with the multivariable regression analysis (Table 4).

As a result of AICc model selection for BCVA3m using the variables of BCVAbase, ΔBCVA1m, MSbase, ΔMS1m, integrity of EZ, the frequency of the injection of anti-VEGF, and age, only BCVAbase, ΔBCVA1m, and ΔMS1m were selected in the optimal model for BCVA3m: BCVA3m = 0.036 + 0.88 x BCVAbase (standard error: SE = 0.085, *p* < 0.001) + 0.76 x ΔBCVA1m (SE = 0.13, p < 0.001) − 0.022 x ΔMS1m (SE = 0.0095, *p* = 0.032): AICc = − 42.1. Removing ΔMS1m from this optimal model resulted in a significant decrease of the loglikelihood (AICc = − 39.8, *p* = 0.033, ANOVA).

As a result of AICc model selection for BCVA3m using the variables of BCVAbase, ΔBCVA1m, qMSbase (instead of MSbase), ΔqMS1m (instead of ΔMS1m), integrity of EZ, the frequency of the injection of anti-VEGF and age, only the optimal model was BCVA3m = 0.041 + 0.90 x BCVAbase (SE = 0.086, *p* < 0.001) + 0.79 x ΔBCVA1m (SE = 0.13, *p* < 0.001) − 0.014 x ΔqMS1m (SE = 0.0070, *p* = 0.053): AICc = − 41.0. Removing ΔqMS1m from this model did not result in a significantly decreased log-likelihood, although it approached a significance (AICc = − 39.8, *p* = 0.053, ANOVA).

## Discussion

In the current study, retinal sensitivity measurement with MP-3 microperimetry was performed in patients with BVO and ME before and after the anti-VEGF treatment, along with OCT measurement. As a result, the change of retinal sensitivity was correlated with retinal volume especially in the affected quadrant; on the other hand, visual acuity was not correlated with retinal structure. Moreover, retinal sensitivity change at 1 month was significantly related to BCVA at 3 months after anti-VEGF treatment, indicating the usefulness of the measurement of retinal sensitivity to predict visual acuity.

This usefulness of the measurement of retinal sensitivity would be further validated by the association with OCT-measured retinal thicknesses; significant relationships were observed between ΔqMS1m and ΔMV1m, ΔqMV1m, and ΔCRT1m whereas this was not the case between ΔBCVA1m and any OCT parameter. Kriechbaum et al. also investigated BCVA, mean retinal sensitivity using MP-1, and OCT-measured retinal volume at 12 months after anti-VEGF treatments in patients with macular edema secondary to retinal vein occlusion [7]. These values were significantly improved compared with baseline, and moreover, BCVA and mean retinal sensitivity were significantly correlated with mean central retinal thickness, but not with retinal volume.

There have been many studies which suggested the usefulness of the anti-VEGF therapy in eyes with BVO with ME [5–8, 15–19]. For instance, Mylonas et al. have reported that BCVA improved significantly at 3 months and 12 months after the anti-VEGF therapy in patients with BVO [15]. Fujihara-Mino et al. also suggested that BCVA significantly improved 1 month after the anti-VEGF therapy in patients with BVO [16]. However, in contrast to these previous studies, in the current study, BCVA did not significantly improve 1 month after anti-VEGF treatment, although the difference approached a significance (*p* = 0.058). This reason for the contradicting result is entirely unknown, but in the current study, BVO patients were consecutively recruited and the majority of patients had already received anti-VEGF treatments prior to the initiation of the study. In contrast to BCVA, MS and qMS significantly improved at 1 month after anti-VEGF treatment, suggesting retinal sensitivity might improve earlier than BCVA.

Sugimoto et al. [8] suggested retinal sensitivity using MAIA at 1 day after the intravitreal bevacizumab was correlated with BCVA at 1 month in patients with BVO. Agreeing with this report [8], our results suggested that the retinal sensitivity at 1 month is a useful predictive factor of BCVA at 3 months, which was consistent with the previous report. More specifically, as a result of model selection, the optimal model for BCVA3m included BCVAbase, ΔBCVA1m, and ΔMS1m or ΔqMS1m. Thus, retinal sensitivity measurement using the MP-3 was useful to precisely predict the prognosis of BCVA.

One of the possible limitations of the current study was that the onset of BVO was not known in all patients, and the period from onset was not included in the analysis. The second limitation of the current study was the exclusion of the eyes with ischemic cases. Manabe et al. reported the association between parafoveal capillary nonperfusion and retinal sensitivity in eyes with resolved BVO patients. They concluded mean retinal sensitivity at the capillary non-perfusion area was significantly lower than that at the capillary perfusion area [20]. It would be of interest that whether retinal sensitivity measurement is also useful in such cases; however, the purpose of the current study was to investigate the usefulness of measuring retinal sensitivity in eyes with BVO and ME, and it was beyond the purpose of the current study. Also, BCVA prognosis was observed up to 3 months, and a longer observation would be needed in a future study.

In conclusion, there was a significant improvement in retinal sensitivity measured with MP-3 microperimetry in BVO patients with ME, whereas no improvement was observed in BCVA at 1 month after anti-VEGF injection. Retinal sensitivity measured with MP-3 was closely related to the OCT-measured structural change after the anti-VEGF treatment, whereas this was not the case with BCVA. The current results suggest the usefulness of the evaluation of the retinal sensitivity in eyes with BVO and ME.
